# Hypoxia-ischemia alters distribution of lysosomal proteins in rat cortex and hippocampus

**DOI:** 10.1242/bio.036723

**Published:** 2018-10-15

**Authors:** M. Troncoso, N. Bannoud, L. Carvelli, J. Asensio, A. Seltzer, M. A. Sosa

**Affiliations:** 1 Laboratorio de Biología y Fisiología Celular “Dr. Franciso Bertini”, Instituto de Histología y Embriología – IHEM-CONICET-FCM-UNCuyo, 5500 Mendoza, Argentina; 2 Facultad de Ciencias Exactas y Naturales, Universidad Nacional de Cuyo, 5500 Mendoza, Argentina

**Keywords:** Hypoxia-ischemia, Excitotoxicity, Lysosomal enzymes, Lysosomes

## Abstract

Neuronal excitotoxicity induced by glutamatergic receptor overstimulation contributes to brain damage. Recent studies have shown that lysosomal membrane permeabilization (LMP) is involved in ischemia-associated neuronal death. In this study we evaluated the effect of neonatal hypoxia-ischemia (HI), as a model of excitotoxicity, on the lysosomal integrity throughout the distribution of the lysosomal proteins cathepsin D and prosaposin. Rat pups (7 days old) of the Wistar Kyoto strain were submitted to HI and they were euthanized 4 days after treatment and the cerebral cortex (Cx) and hippocampus (HIP) were processed for immunohistochemistry or immunoblotting. Treatment induced an increase of gliosis and also a redistribution of both prosaposin and cathepsin D (as intermediate and mature forms), into the cytosol of the HIP and Cx. In addition, HI induced a decrease of LAMP-1 in the membranous fraction and the appearance of a reactive band to anti-LAMP-1 in the cytosolic fraction, suggesting a cleavage of this protein. From these results, we propose that the abnormal release of Cat D and PSAP to the cytosol is triggered as a result of LAMP-1 cleavage in HI animals, which leads to cell damage. This could be a common mechanism in pathological conditions that compromises neuronal survival and brain function.

## Abstract (precis)

**Summary:** Hypoxia ischemia (HI) induces an increase of gliosis and redistribution of prosaposin and cathepsin D into the cytosol of rat hippocampus. This could be triggered by LAMP-1 cleavage in HI.

## INTRODUCTION

The hypoxia-ischemia (HI) excitotoxic injury model in the immature brain has been widely used to study the mechanisms of neuronal injury and endogenous neuronal repair (Vannucci et al., 1995; López-Aguilera et al., 2012; Rumajogee et al., 2016). Damage to the central nervous system (CNS) leads to cellular changes not only in the affected neurons but also in adjacent glial cells and endothelia. An increased immune chemically-detectable GFAP level was one of the earliest responses to specifically characterize CNS injuries. Hypertrophic astrocytes seem to be an unspecific population present in a large variety of distinct nerve pathological entities in experimental animal models. Dysfunctional lysosomes seem to be associated to reactive astrocytes which ultimately lead to the onset of cell death (reviewed by Castejón, 2015). The HI brain injury has drastic molecular consequences derived from energy deficit.

This complex process involves glutamate release, followed by activation of glutamate receptors, activation of the enzyme nitric oxide synthase, calcium influx, release of nitric oxide and a consequent mitochondrial dysfunction (Rocha-Ferreira and Hristova, 2016). The lysosomal destabilization by oxidative stress and other apoptotic signals and subsequent cathepsin D leakage to the cytosol can induce apoptotic changes of mitochondria and eventually cell death. A series of studies have shown the destabilization of the lysosomal membrane to be an early event in cellular devitalization (Öllinger and Brunk, 1995; Roberg et al., 1999). In developing brains, the HI lesion causes both early and delayed neurodegeneration, and the type of neuronal death (apoptosis or necrosis) is determined by the time elapsed after the injury, by the affected brain area, and potentially, by patterns of neural connectivity (Northington et al., 2001).

In recent years, special attention has been paid to the study of the function of the endosomal–lysosomal system in the nervous system and its close relationship with the process of cell death. Lysosomes are cytoplasmic membrane-enclosed organelles that contain hydrolytic enzymes that participate in the control of the intracellular turnover of macromolecules (Luzio et al., 2007). These organelles contain many different types of hydrolytic enzymes that usually exert their maximal enzymatic activity at a low pH. The high concentration of hydrolytic enzymes is potentially harmful for cell survival (Boya and Kroemer, 2008). The lysosomal membrane is protected from the acidic hydrolases by the specific expression of membrane proteins such as LAMP-1 (lysosomal-associated membrane protein 1) and LAMP-2 (lysosomal-associated membrane protein 2), which are highly glycosylated and therefore, they resist digestion (Eskelinen, 2006). It is well known that the total rupture of lysosomes can produce a cytosolic acidification with leakage of hydrolases and cell death by necrosis, while a partial and selective lysosomal rupture can lead to cell death by apoptosis (Terman et al., 2006a,b). The redistribution of soluble lysosomal components (including enzymes) from the lumen to the cytosol is the distinctive feature for lysosomal membrane permeabilization (Boya et al., 2003). More recently, the permeabilization of the lysosomal membrane has been associated with the development of neurodegenerative diseases such as Alzheimer's, Parkinson's and Huntington's (Boya and Kroemer, 2008; Dehay et al., 2010; Micsenyi et al., 2013).

The hydrolytic enzyme cathepsin D (Cat D) is an endolysosomal aspartic protease, synthesized as a proenzyme and delivered to late endosomes by mannose-6-phosphate receptors. In the late endosomes, a proteolytic cleavage of the N-terminal propeptide produces the 48 kDa intermediate form, which is further processed to generate the mature enzyme, which comprises two fragments of 34 and 14 kDa (Stoka et al., 2016). In several animal models, it has been proved that an excessive Cat D proteolytic activity is cytotoxic, since the enzyme is released into the cytosol, where it triggers mechanisms that lead to cell death (Shacka et al., 2007; Follo et al., 2011). Prosaposin (PSAP) is another widely studied lysosomal protein that is expressed in several tissues, such as the brain, the skeletal muscle and the heart (Sano et al., 1989; Terashita et al., 2007). PSAP is the precursor of four lysosomal glycoproteins: saposins A–D (Kishimoto et al., 1992; Meyer et al., 2014), which activate lysosomal hydrolases and the synthesis of specific sphingolipids (O'Brien et al., 1988; Kishimoto et al., 1992). Several studies have proposed that PSAP is a neurotrophic factor that prevents neuronal death and promotes neurite enlargement in different types of neurons (O'Brien et al., 1994). In addition, this protein has shown a mielinotrophic action on Schwann cells and oligodendrocytes by increasing the sulfatide content and preventing glial death (Hiraiwa et al., 1997; Campana et al., 1998).

Given that the endosomal–lysosomal system plays an important role in the homeostasis and nervous tissue integrity, and based on previous studies, the aim of this study was to evaluate the effect of HI on the lysosomal integrity throughout the distribution of lysosomal proteins. To this end, we evaluated expression and distribution of some lysosomal proteins, such as Cat D, PSAP and glycosidases, in the cerebral cortex (Cx) and the hippocampus (HIP) of HI rats. We also attempted to establish a link between HI and gliosis in the brain areas under study, as an indicator of neuronal injury. In addition, a relationship between these proteins and the lysosomal integrity throughout the status of the membrane protein LAMP-1 was also evaluated.

## RESULTS

### The HI injury induces astrogliosis

**Fig. 1.**
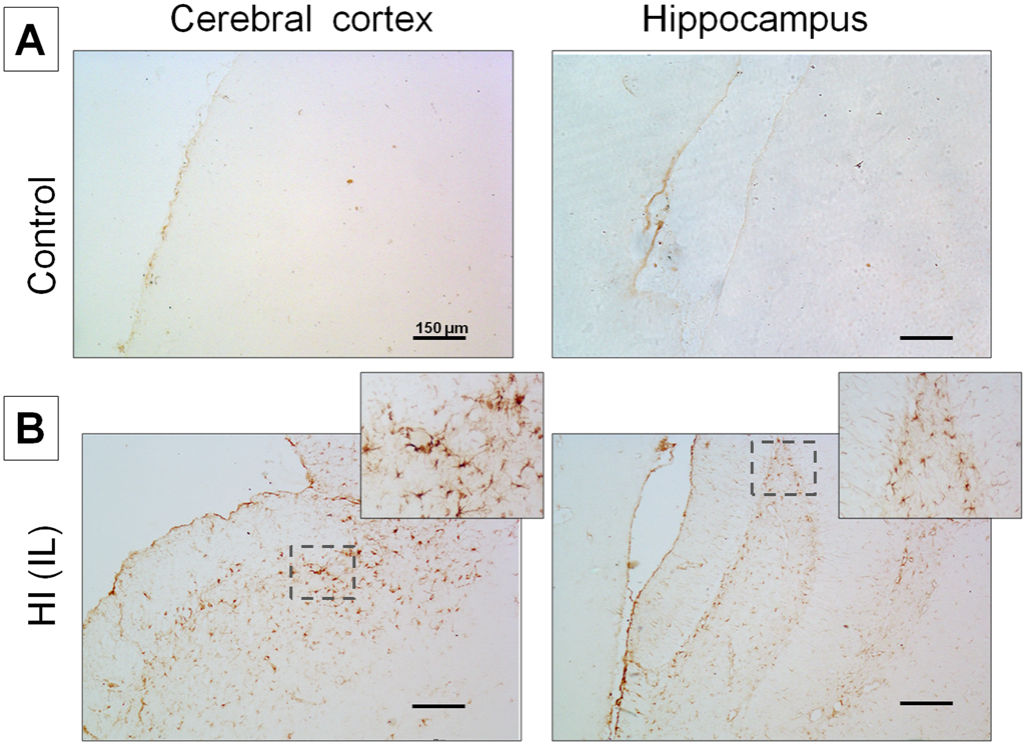
**Detection of astrogliosis employing** **an anti-GFAP antibody in normal and injured brains by HI.** (A) Control Cx and HIP; (B) Ipsilateral (IL HI) Cx and HIP subjected to HI. A,B: 10× magnification. Image B shows the amplification of the area indicated by dashed lines (20× magnification). Scale bars: 150 μm.

As a first approach we confirmed the presence of reactive astrocytes in the injured tissue caused by the HI procedure. Among glial cells, the astrocytes perform various physiological functions and also contribute to the inflammation associated with excitotoxicity and cell injury. We observed abundant labeled cells with thick short processes characteristic for reactive astrocytes in both brain areas under study when tissue was immuno-probed with GFAP (a classical marker of intermediate filaments) in the Cx and HIP belonging to animals injured by HI, as compared to non-injured control brains (Fig. 1).

### Intermediate and mature forms of the lysosomal enzyme Cat D increase in the cytosolic fraction of cortex and hippocampus of HI animals

Since the endosomal–lysosomal system plays an important role in the homeostasis and neuronal integrity, the effect of neonatal HI on the compartmentalization of some lysosomal enzymes and proteins that participate in the nervous cells metabolism was studied. This study focused on Cat D, PSAP and glycosidases which are known to be involved in excitotoxic and neurodegenerative processes.

**Fig. 2.**
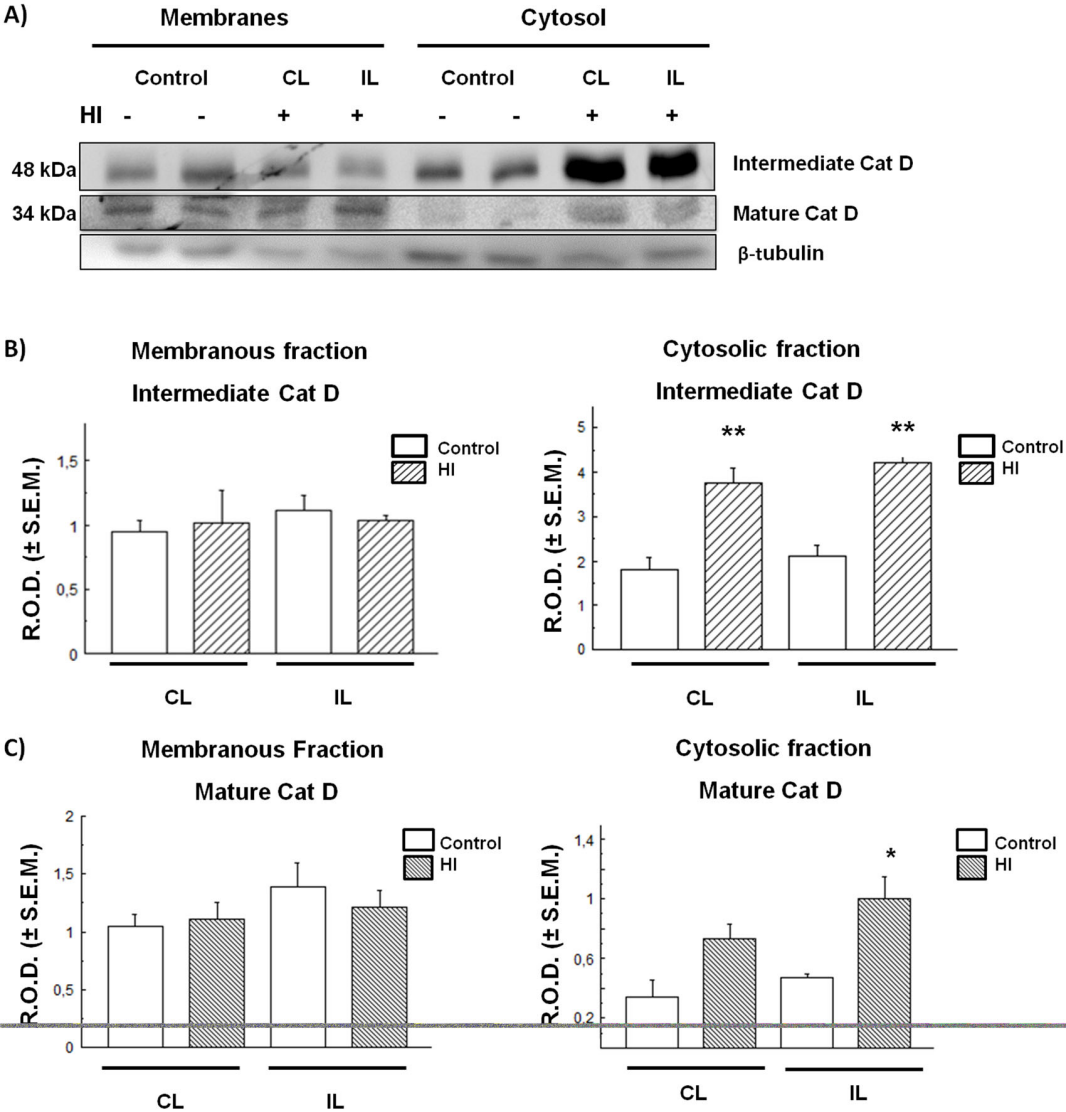
**CatD expression and distribution in control and HI rat cerebral cortex.** (A) Immunodetection of Cat D in membrane and cytosol. (B,C) Densitometric band quantification corresponding to intermediate and mature forms, respectively. Bars represent the mean of relative optical densities (R.O.D.±s.e.m.) from three independent experiments. *P<0.05, **P<0.01, as compared to controls. IL, ipsilateral; CL, contralateral brain hemisphere. β-tubulin was used as loading control.

**Fig. 3.**
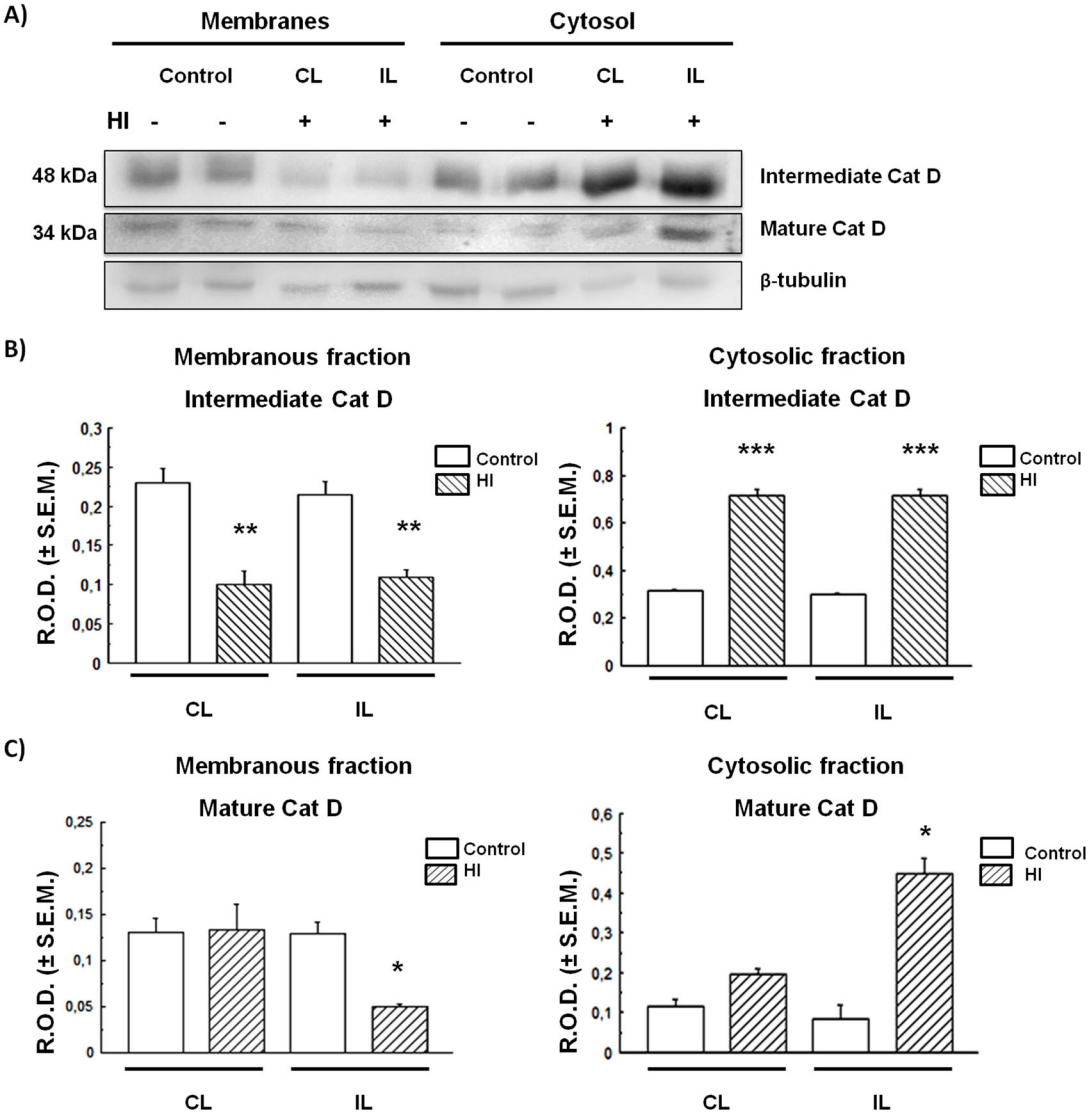
**CatD expression and distribution in control and HI rat hippocampus.** (A) Immunodetection of Cat D in membrane and soluble fractions. (B,C) Densitometric quantification of bands corresponding to intermediate and mature Cat D forms respectively. Bars represent the mean of relative optical densities (R.O.D.±s.e.m.) from three independent experiments. *P<0.05, **P<0.01, ***P<0.001, as compared to controls. IL, ipsilateral; CL, contralateral brain hemisphere. β-tubulin was used as loading control.

The Cx and HIP were separated into cytosolic (soluble) and membranous fractions by centrifugation at 50,000× ***g***, and Cat D levels were evaluated in each fraction. As shown in Fig. 2, Cat D intermediate (48 kDa) and mature (34 kDa) forms were detected in the Cx. Although no differences between control and HI animals were found in the membranous fraction of this brain area, the levels of both Cat D forms were found to be significantly increased in the HI Cx cytosolic (soluble) fraction with respect to controls (Fig. 2B,C). This could indicate an apparent increase in Cat D expression in the Cx with a release of the excessive enzyme to the cytosol in HI rats. Unlike the Cx in the HI HIP, the detection of both the intermediate and mature forms of Cat D decreased considerably in the membranous fraction, whereas they were increased in the cytosolic fraction, as compared to controls (Fig. 3).

### HI induces redistribution of prosaposin in the rat cortex and hippocampus

**Fig. 4.**
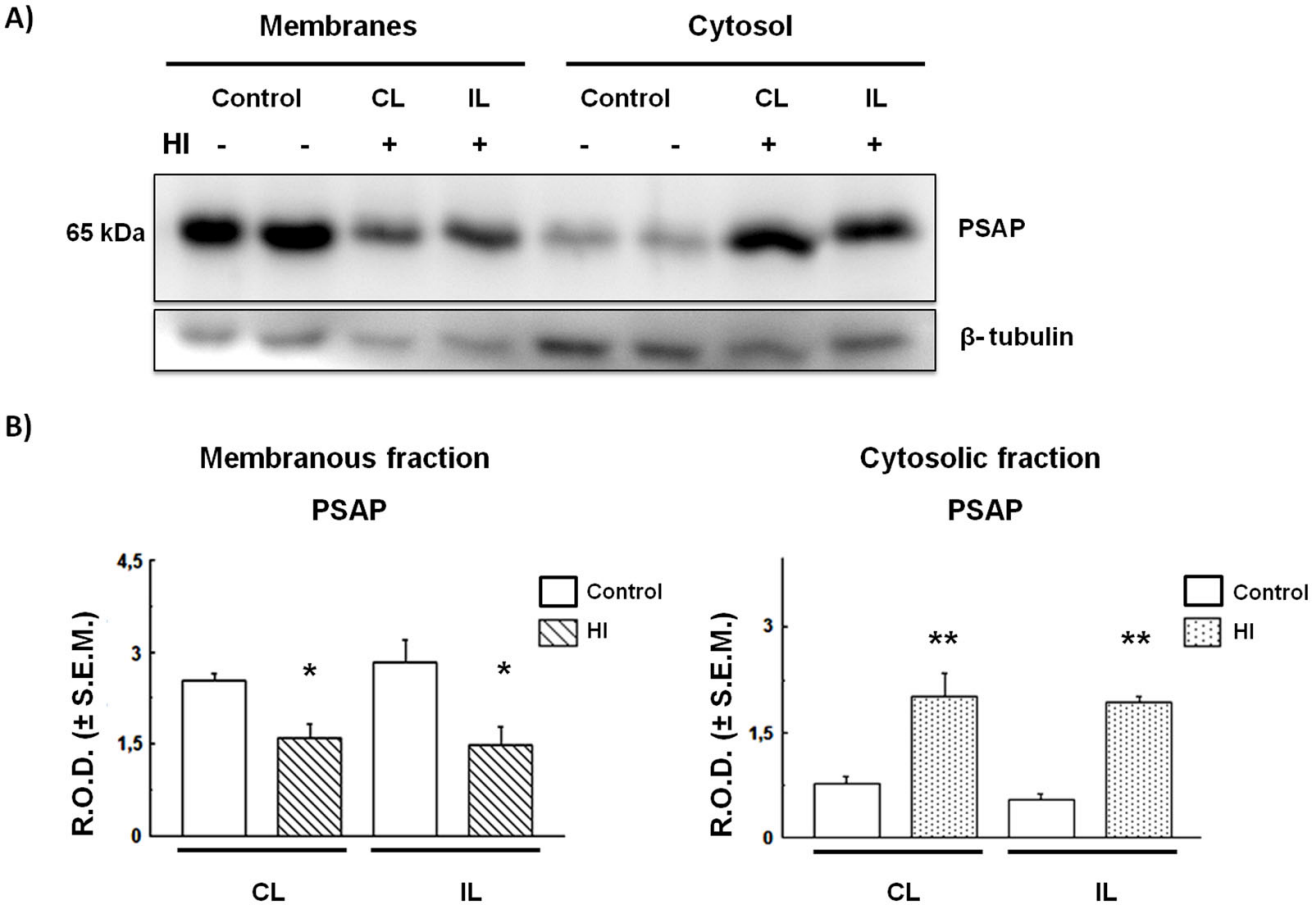
**PSAP expression and distribution in control and HI rat cerebral cortex.** (A) Immunodetection of PSAP in membrane and cytosolic fractions. The same membrane from Fig. 2 was stripped as detailed in the Materials and Methods section and processed for PSAP or tubulin detection. (B) Densitometric quantification of the bands obtained in A. Bars represent the mean of relative optical densities (R.O.D.±s.e.m.) from three independent experiments.*P<0.05, **P<0.01, as compared to controls. IL, ipsilateral; CL, contralateral brain hemisphere. β-tubulin was used as loading control.

**Fig. 5.**
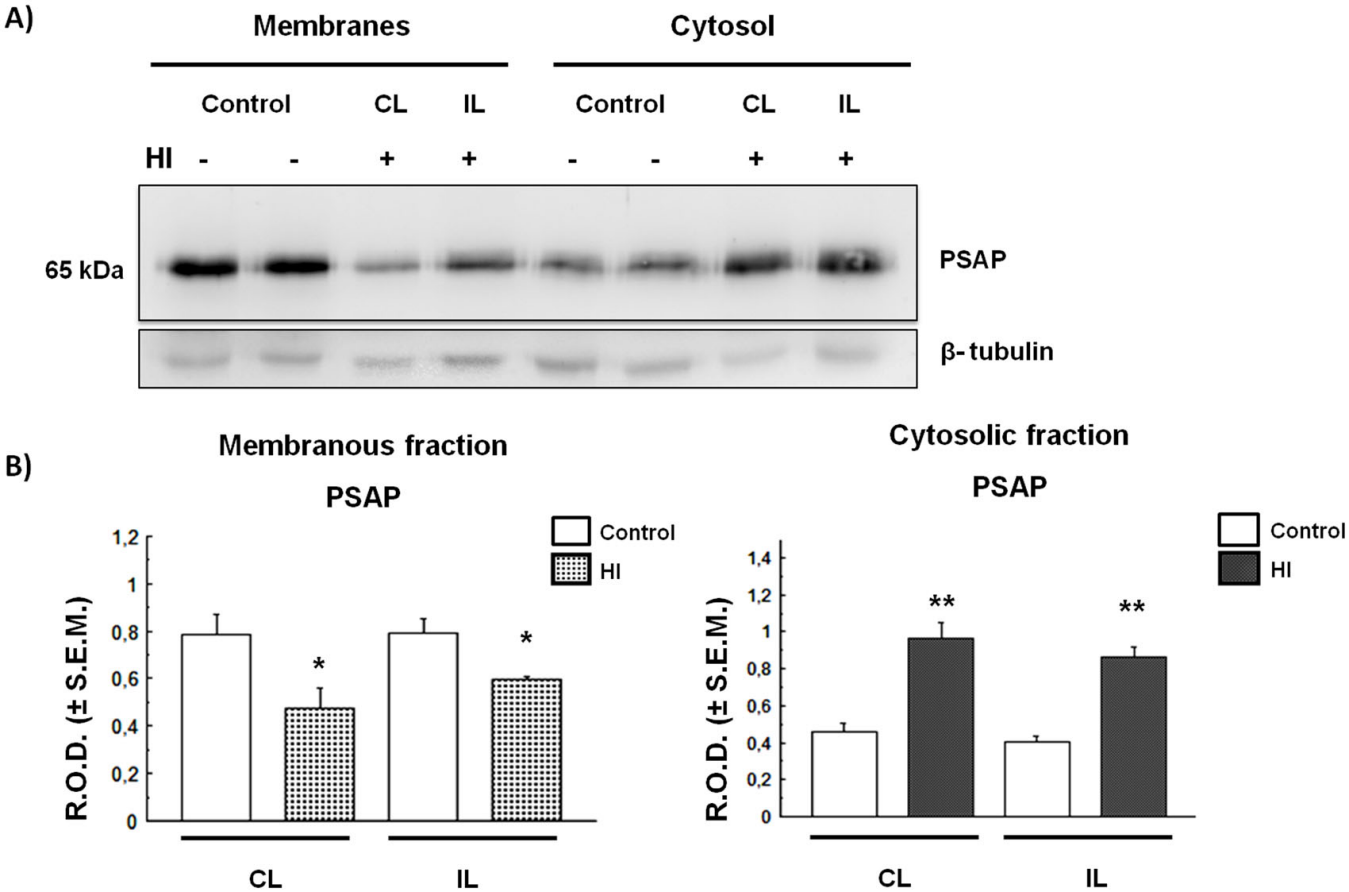
**PSAP expression and distribution in HI rat hippocampus.** (A) Immunodetection of PSAP in membrane and cytosolic fractions. The same membrane from Fig. 3 was stripped as detailed in the Materials and Methods section and processed for PSAP or tubulin detection. (B) Densitometric quantification of the bands obtained in A. Bars represent the mean of relative optical densities (R.O.D.±s.e.m.) from three independent experiments. *P<0.05, **P<0.01, as compared to controls. IL, ipsilateral; CL, contralateral hemisphere. β-tubulin was used as loading control.

As with Cat D, the PSAP distribution was studied in the Cx and HIP of HI rats. As shown in Fig. 4, the PSAP levels increased significantly in the cytosolic fraction as they decreased in the membranous fraction obtained from HI Cx, as compared to controls. These changes were observed in both CL and IL hemispheres of HI rats. As occurred in the cortex, PSAP levels in the cytosolic fraction increased significantly in both IL and CL horns of HI hippocampus, as compared to controls. This increase was also at the expense of a decrease in the membranous fraction (Fig. 5). The increase of PSAP in the cytosolic fraction obtained from the cortex and hippocampus of HI rats suggests that these proteins are released from the membranous compartments to the cytosol. This process could be due to a permeabilization (partial or total) of membrane-bound compartments as a consequence of HI injury. In order to clarify this issue, we evaluated the status and compartmentalization of LAMP-1, a lysosomal-associated membrane protein.

### HI induces cleavage of LAMP-1 in the cortex and hippocampus

**Fig. 6.**
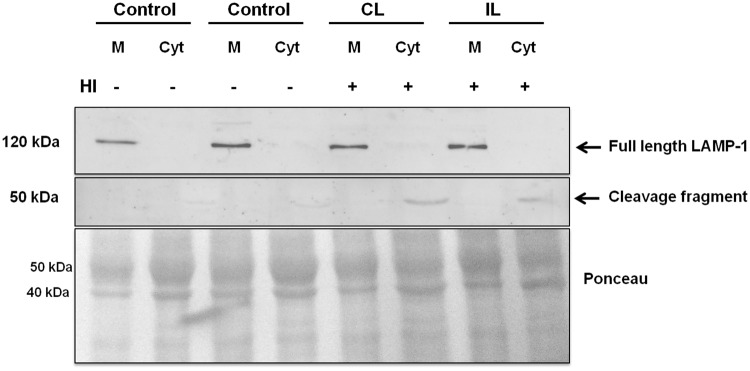
**Effect of HI on the integrity of LAMP-1.** Immunodetection of LAMP-1 in membranes and cytosol of control and HI Cx. Ponceau Red protein staining was used as loading control. A representative image of two independent experiments is shown. IL, ipsilateral; CL, contralateral brain hemisphere; M, membranes; Cyt, Cytosol.

**Fig. 7.**
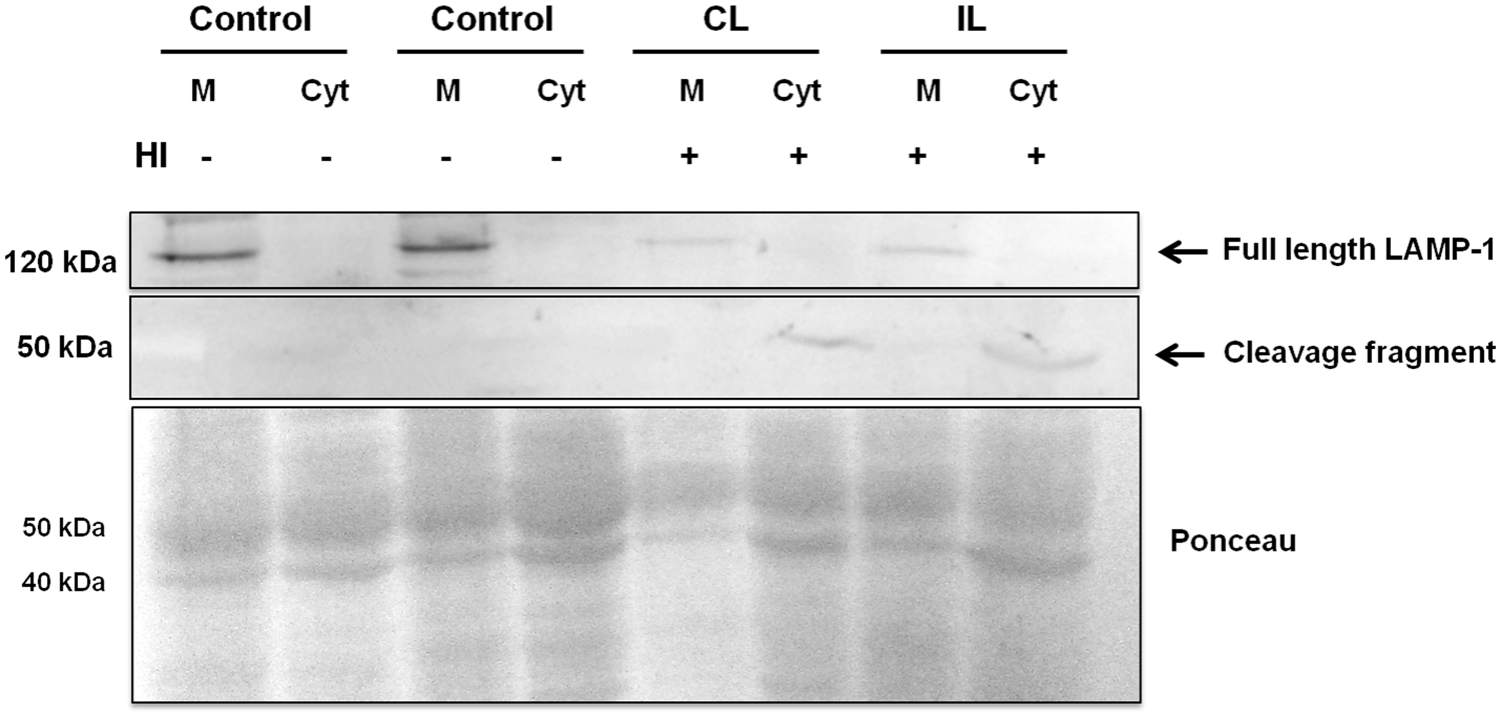
**Effect of HI on the integrity of LAMP-1 protein.** Immunodetection of LAMP-1 in membranes and cytosol from control and HI hippocampus. Ponceau Red staining was used as loading control. A representative image of two independent experiments is shown. IL, ipsilateral; CL, contralateral brain hemisphere; M, membranes; Cyt, Cytosol.

Highly-glycosylated transmembrane proteins play an important role in the maintenance of lysosomal integrity. Damage to organelle–membrane components or changes in their structure and fluidity may lead to lysosomal destabilization, with the consequent leakage of lysosomal enzymes and proteins to the cytosol. Based on our results, we analyzed the LAMP-1 expression in the Cx and HIP of control and HI rats. LAMP-1 (120 kDa band) is expressed in the membranous fraction from control and HI groups, in both brain areas (Figs 6,7). However, the expression of LAMP-1 decreased in the membranous fraction from HI HIP, (Fig. 6), while a 50 kDa band appeared in the cytosolic fraction of the brain areas from both hemispheres of HI animals. This component might correspond to a fragment resulting from LAMP-1 cleavage (Figs 6,7). Although a 50 kDa fragment was also observed in the cytosolic fraction of HI hippocampus, the expression of the whole 120 kDa protein did not decrease in the membranous fraction.

### Compartmentalization of other acidic hydrolases

**Fig. 8.**
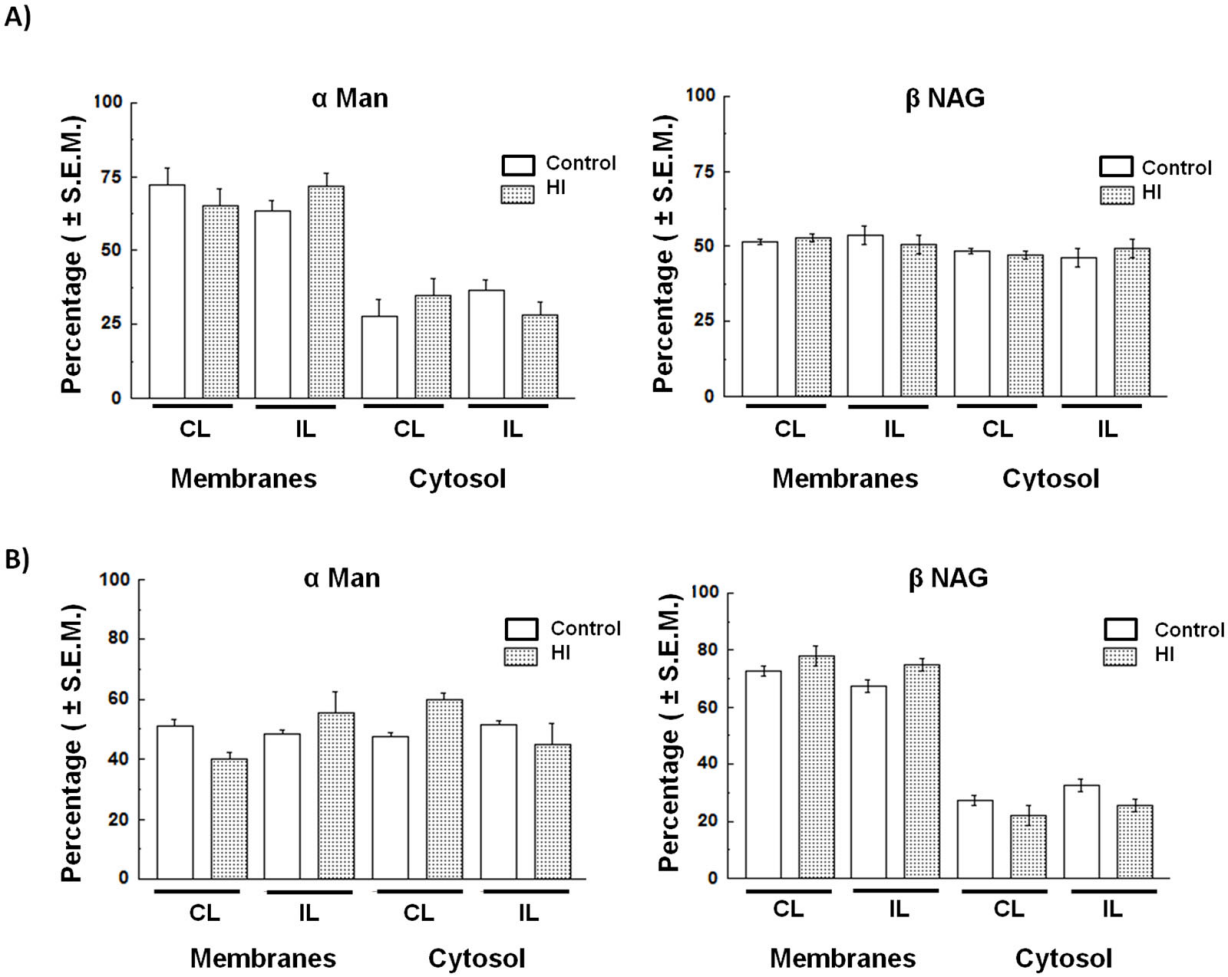
**Distribution of αMan and βNAG enzyme activity.** (A) In the cerebral cortex (Cx) and (B) in the hippocampus (HIP). Values are expressed as percentage (mean) of specific enzymatic activity (U/mg protein)±s.e.m. from three independent experiments. IL, ipsilateral; CL, contralateral brain hemisphere; αMan, α-mannosidase; βNAG, N-acetyl β-D-glucosaminidase.

In order to determine if the phenomenon observed in the HI Cx and HIP is a general mechanism for all lysosomal proteins, we also studied the distribution of other acid hydrolases, such as α-mannosidase (α Man) and β-N-acetylglucosaminidase (βNAG). No significant differences were found in the enzymatic activity in neither the Cx nor the HIP from control and HI rats (Fig. 8).

## DISCUSSION

In this study we described some molecular changes occurring in two rat brain areas as responses to the excitotoxicity induced by HI. Astrocyte proliferation and increased GFAP levels, are indicators of reactive astrogliosis (Sofroniew and Vinters, 2010), widely used as a pathological hallmark of altered CNS tissue (Rodríguez et al., 2009; Sofroniew and Vinters, 2010; Robel et al., 2015; Sofroniew, 2015). In order to validate our model, we evaluated the increase of GFAP labeling in the brains of the animals submitted to HI to demonstrate the presence of reactive astrocytes, as compared to controls. It is known that astrocytes are sensitive to glutamate excitotoxicity in response to ischemia, however, the role of reactive glia is still controversial, as to whether these cells perform neuroprotective or detrimental actions.

As mentioned above, excitotoxicity is considered one of the most important pathological mechanisms mediating the loss of neurons in the CNS, in both acute and chronic diseases. Excitotoxicity activates mechanisms that are, in many cases, irreversible, such as the increase of protease activity, i.e. calpains, whose proteolytic function contributes to neuronal death (Yan et al., 2016). In addition, severe rat neonatal HI enhances autophagy as mechanism of excitotoxicity, triggering apoptosis in the cortex but not in other brain areas (Ginet et al., 2009). It has recently been reported that lysosomal permeabilization is associated with an early stage of glutamate-mediated excitotoxicity in primary cultures of neurons (Yan et al., 2016). Neurons are not the only cells affected by excitotoxicity, as astrocytes and endothelial cells in the brain tissue are also targets for degradation of cytoplasmic macromolecules and organelles via the lysosomal system (Xu and Zhang, 2011). In addition, in other models of focal ischemia (in rodents and primates) an increase of cathepsins in the cytosol was found as a result of lysosomal permeabilization (Yamashima et al., 1998, 2003). In this work we found an increase in the intermediate and mature forms of Cat D in the cytosolic fraction of Cx (Fig. 1B,C) and HIP (Fig. 2B,C) in HI rats. This finding suggests that there is an abnormal leakage of these enzymes from the lysosomes to the cytosol, either as a cause or consequence of the excitotoxic damage. Such increase of Cat D in the cytosol could be the result of a partial or total permeability of the lysosomal membrane. Many studies have shown that different excitotoxic stimuli can lead to an increase in the activity of calpains, calcium-dependent proteases, and to the generation of reactive oxygen species that promote the lysosomal membrane destabilization during neuronal damage. Examples of such stimuli may be transient focal ischemia, glucose and oxygen deprivation, or global ischemia (Yamashima et al., 1996, 1998; Yap et al., 2006).

Moreover, we found that the lysosomal protein PSAP increases in the Cx and in the HIP cytosol of HI animals (Figs 4,5). This increment, apparently, is at the expense of a decrease in the amount of protein associated with the membranous fraction (lysosome-endosome) and also suggests the existence of a shift in PSAP localization from the endo-lysosomal compartments towards the cytosol. These effects are similar to those observed for Cat D (Figs 2,3), suggesting the existence of a disruption of the lysosomal compartment in HI animals. Although PSAP has been proposed as a neuroprotective protein (Meyer et al., 2014), the cytosolic increment that we describe could be related to a protein redistribution and not to a change in its expression.

Interestingly, no differences were found in the distribution of other acid hydrolases, such as αMan and βNAG, between the experimental groups (Fig. 8). This observation could be explained by the existence of several lysosomal populations, as suggested by other authors, each of which could contain various sets of enzymes with different sensitivities to injury (Colombo and Bertini, 1985; Cuervo et al., 1997). In addition, the lysosomal damage could derive from a partial permeabilization, which would not necessarily occur in all lysosomal populations.

The lysosomal membrane stability and lysosome integrity depend on the high glycosylation rate of transmembrane proteins that protect them from autodigestion (Eskelinen, 2006). Among these proteins, LAMP-1 and LAMP-2 have been widely studied. It was of interest to assess whether changes in LAMP-1 were responsible for the lysosome permeabilization occurring in HI animals. We observed in the cytosolic fraction of the HI brain a 50 kDa band when probed with the anti-LAMP-1 antibody. We suggest that this band is present as the result of a cleavage of the 120 kDa LAMP-1 protein in response to HI, since its appearance was concomitant to the loss of the whole protein in the HI hippocampus. Furthermore, it has been reported that LAMP-1 is sensitive to protease activity (Lin et al., 1997). Thus, it can be hypothesized that the cleavage of LAMP-1 would be the result of the activation of calpain caused by a calcium influx. Taking these findings into account, we interpret that the abnormal release of Cat D and PSAP to the cytosol is triggered as a result of LAMP-1 cleavage in HI, which leads to cell damage. This could be proposed as one of the many possible mechanisms in pathologic states that compromise the neuronal function and survival.

## MATERIALS AND METHODS

### Antibodies

A goat anti-cathepsin D antiserum was purchased from Santa Cruz Biotechnology (sc-6487 Dallas, USA). Rabbit anti-prosaposin antiserum was kindly provided by Dr Morales (Mc Gill University, Montreal, Canada) (Lefrancois et al., 2003). The rabbit anti-LAMP-1 (ab-24170) antiserum and anti-tubulin monoclonal antibody (ab-56676) were obtained from Abcam (USA), anti-Glial-Fibrillary Acidic Protein (GFAP) monoclonal antibody produced in mouse was obtained from Sigma-Aldrich (G-3893, USA). HRP-conjugated anti-goat IgG antiserum was obtained from H&L (401515), the HRP-conjugated anti-rabbit IgG fraction of antiserum was obtained from Sigma-Aldrich (A 9169) and anti-mouse IgG (whole molecule)-peroxidase was purchased from Sigma-Aldrich (A 9044). Chemiluminescent reagents were obtained from Pearce (Rockford, USA).

### Animals

Rat pups (7 days old) of the Wistar Kyoto (WKY) strain (*n*=12) were used for this study. Animals were maintained with their mothers under a light cycle of 12L: 12D and controlled temperature (23±1°C) and humidity. All experimental procedures were carried out in accordance with the regulations of the National Institute of Health Guide for the Care and Use of Laboratory Animals and by the local animal-ethic instructions established by CICUAL (*Comité Institucional para el Cuidado y Uso de Animales de Laboratorio, of the Facultad de Ciencias Médicas, Universidad Nacional de Cuyo*: Aval no. 35/2014) Mendoza, Argentina.

### HI experiments

Unilateral common carotid artery ligation was performed on 6 animals under isoflurane anesthesia. The left common carotid artery was ligated at two points through a midline cervical incision. Following surgery, rats were returned to the cages with their mothers and littermates for 2 h, and then they were placed in an air-tight chamber containing 100% N_2_ at 37°C for 1–3 min until they showed signs of asphyxia. After recovering the normal breathing, animals were returned to their cages. Control animals (*n*=6) were also subjected to surgery but without carotid ligation or subsequent exposure to the N_2_ atmosphere. All animals were euthanized by decapitation on day 4 after ligation (11 days old). Some animals (3 from controls and 3 from HI experimental groups respectively) were used for western blot analysis (each animal was processed separately). In addition, another 3 animals from both experimental groups were used for immunohistochemical analyses.

### Processing of biological material for immunoblotting

Control and HI rats were euthanized by decapitation and their brains were rapidly removed and dissected on a cold plate. Cortex and hippocampus were obtained and placed into individual tubes containing homogenization buffer (20 mM HEPES buffer pH 7.4, 0.25 M sucrose, 2 mM EDTA, 1 mM PMSF, 10 mM NaF, 1 µM pepstatin A, 100 µM leupeptin, 5 mM sodium glycerophosphate and 1 mM sodium *o-*vanadate). Tissues were homogenized individually with a teflon pestle homogenizer and then centrifuged at 800× ***g*** for 10 min. Post-nuclear supernatants were subjected to further centrifugation at 50,000× ***g*** for 30 min to obtain the membranous and cytosolic fractions from each tissue. Samples were stored at −20°C until used.

### Immunoblotting

Equivalent quantities (40 µg) of membrane or cytosolic proteins from each sample were boiled in SDS sample buffer (Laemmli, 1970), and analyzed by 10% SDS-PAGE. After electrophoresis, proteins were electrotransferred to nitrocellulose blotting membranes (GE Healthcare, Amersham, Germany). Membranes were blocked with 3% skimmed milk in PBS for 1 h and then incubated overnight with antibodies against Cat D (1:1000) or LAMP-1 (1:1000) all diluted in PBS-T [10 mM NaH_2_PO_4_-Na_2_HPO_4_, pH 7.2, PBS, containing 0.05% (v/v) Tween 20]. Membranes were then washed twice with PBS-T and incubated for 2 h with the corresponding HRP-conjugated secondary antibodies. For PSAP detection, the same membrane used for Cat D in each experiment was stripped as follows: the membrane was washed with stripping solution (2 mM glycine pH 2.5, containing 0.05% Tween 20) during 30 min at room temperature and then washed 3 times with PBS-T (10 min each). After washings, the membrane was blocked with skimmed milk as before and then incubated overnight with anti-PSAP (1:5000), followed by the corresponding secondary antibody. In all cases, chemiluminescent signal was detected with a LAS 4000 imaging system (Fujifilm Lifescience, USA). Bands were quantified by densitometry using the ImageJ software (Image Processing and Analysis in Java; National Institutes of Health). Detection of β-tubulin (after membrane stripping) or Ponceau Red was used as loading control.

### Immunohistochemistry

Brains were removed and immediately fixed in Zamboni solution for 12 h. The different brain areas were then dehydrated and embedded in paraffin using standard procedures. Five µm-thick sections were obtained starting along the mid-sagittal axis. Microscopy slides were deparaffinized in xylene and rehydrated by washing in decreasing concentrations of ethanol and then immersed in 0.1 M citrate buffer (pH: 6) at 96°C during 15 min for antigen retrieval. The non-specific binding was blocked with 5% BSA-PBS for 30 min in a humid chamber at room temperature. Tissue sections were incubated overnight at 4°C with the primary antibody (GFAP 1:500). The following day, after washing 3 times (5 min each) in PBS, slides were incubated with an anti-mouse IgG conjugated to biotin (Sigma A9044) (1:200) for 2 h, and then with ExtrAvidin peroxidase (Sigma E2886) for 30 min at room temperature. Protein expression was visualized using 3, 3′-diaminobenzidine staining (DAB, Sigma-Aldrich). The reaction was stopped with phosphate buffer (PB), rinsed in distilled water, dehydrated and mounted with DPX mounting medium. Images were obtained with a conventional light microscope (Nikon 80i) coupled to a digital camera.

### Other procedures

The activity of αMan and βNAG was measured fluorometrically, using the corresponding 4-methyl-umbellyferyl substrates as described by Barrett and Heath (1977). One unit of enzymatic activity corresponded to 1 nmol of substrate digested per hour of incubation. Proteins were measured according to Lowry et al. (1951).

### Statistics

Data from the different groups were analyzed with the Student's *t*-test and the level of significance was set at *P*<0.05.
